# IAP antagonists sensitize murine osteosarcoma cells to killing by TNFα

**DOI:** 10.18632/oncotarget.8980

**Published:** 2016-04-25

**Authors:** Tanmay M. Shekhar, Mark A. Miles, Ankita Gupte, Scott Taylor, Brianna Tascone, Carl R. Walkley, Christine J. Hawkins

**Affiliations:** ^1^ Department of Biochemistry and Genetics, La Trobe Institute for Molecular Science, La Trobe University, Bundoora, Victoria, Australia; ^2^ St. Vincent's Institute of Medical Research, Fitzroy, Australia; Department of Medicine, St. Vincent's Hospital, University of Melbourne, Fitzroy, Australia

**Keywords:** IAP, Smac, bone cancer, RIP1, osteosarcoma

## Abstract

Outcomes for patients diagnosed with the bone cancer osteosarcoma have not improved significantly in the last four decades. Only around 60% of patients and about a quarter of those with metastatic disease survive for more than five years. Although DNA-damaging chemotherapy drugs can be effective, they can provoke serious or fatal adverse effects including cardiotoxicity and therapy-related cancers. Better and safer treatments are therefore needed. We investigated the anti-osteosarcoma activity of IAP antagonists (also known as Smac mimetics) using cells from primary and metastatic osteosarcomas that arose spontaneously in mice engineered to lack p53 and Rb expression in osteoblast-derived cells. The IAP antagonists SM-164, GDC-0152 and LCL161, which efficiently target XIAP and cIAPs, sensitized cells from most osteosarcomas to killing by low levels of TNFα but not TRAIL. RIPK1 expression levels and activity correlated with sensitivity. RIPK3 levels varied considerably between tumors and RIPK3 was not required for IAP antagonism to sensitize osteosarcoma cells to TNFα. IAP antagonists, including SM-164, lacked mutagenic activity. These data suggest that drugs targeting XIAP and cIAP1/2 may be effective for osteosarcoma patients whose tumors express abundant RIPK1 and contain high levels of TNFα, and would be unlikely to provoke therapy-induced cancers in osteosarcoma survivors.

## INTRODUCTION

The primary bone cancer osteosarcoma typically arises in teenagers, with an incidence of 8 per million per year in adolescents and young adults. Osteosarcoma is rare in people aged 25-60, but its incidence increases in older individuals including those with Paget's disease [1]. The introduction of high dose multi-agent chemotherapy in the 1970s, particularly doxorubicin and cisplatin, led to substantial increases in survival rates for osteosarcoma patients [2]. Unfortunately, little recent progress has been made. Around 40% of patients diagnosed with osteosarcoma, and almost three quarters of those with metastatic disease, still die within five years of diagnosis [2].

Almost 90% of the individuals who survive osteosarcoma suffer from disease and/or disability due to their treatment [3]. Most osteosarcoma patients are treated with high dose methotrexate, doxorubicin and cisplatin, before and after surgery [4]. These chemotherapeutic agents can cause severe and sometimes fatal adverse effects including cardiac and kidney damage, neurotoxicity and/or therapy-related second cancers [5], which preclude administration of higher, more effective, doses of these drugs. Around 1 in 13 osteosarcoma survivors develop subsequent malignancies within 30 years of diagnosis [6], more than half of whom die of their second cancers within five years [6]. To improve outcomes for osteosarcoma patients, drugs are needed that can either (1) eliminate osteosarcoma cells as sole agents, without causing severe sequelae, or (2) cooperate with lower doses of currently used chemotherapies to destroy tumor cells more safely.

A number of anti-cancer agents have been recently developed that directly engage programed cell death pathways, rather than initially provoking DNA damage in order to secondarily stimulate tumor cell death. One class of new agents acts by antagonizing members of the IAP family of pro-survival proteins. One member of this family, XIAP, inhibits caspases-3, -7 and -9 to block apoptosis [7]. Two other relatives, cIAP1 and cIAP2, are ineffective caspase inhibitors [8]. Instead, these proteins participate in a complex assembled around the cytoplasmic domain of TNF-R1 that promotes canonical NF-κB-mediated expression of proteins with roles in pro-cancerous process including proliferation, invasion and inflammation [9-11]. cIAP1 and 2 were reported to be frequently overexpressed in osteosarcomas and their amplification-mediated upregulation drove osteosarcomagenesis in p53 heterozygous mice [12]. Crucially, cIAP1/2 can poly-ubiquitinate the kinase RIPK1, preventing it from transitioning into secondary complexes, the “ripoptosome” and “necrosome”, that initiate caspase-dependent apoptotic or caspase-independent necroptotic cell death respectively [13].

IAP activity within cells can be reduced by Smac/Diablo, a natural IAP antagonist protein [14, 15]. A number of “IAP antagonists” (also known as “Smac mimetics”) have been created that bind IAPs in a similar way to Smac, relieving XIAP-mediated caspase inhibition and promoting the proteosomal degradation of cIAP1 and 2 [16]. This loss of cIAP1/2 results in CYLD-mediated deubiquitination of RIPK1, leading to formation of the ripoptosome [17]. Caspase-8 is activated within this complex, and can then proteolytically activate caspases-3 and -7. If the IAP antagonist drug prevents XIAP from inhibiting these executioner caspases, they can induce apoptosis. RIPK1 can also promote caspase-independent, necroptotic cell death [18, 19]. This pathway classically involves a relative of RIPK1, RIPK3, and the pseudokinase MLKL [20, 21]. Necroptosis is negatively regulated by caspase-8 [22] and can be provoked by TNFα treatment of cells lacking IAP and caspase activity.

IAP antagonist drugs can be sub-classified by their structures and specificities. “Monovalent” IAP antagonists such as Debio1143(AT-406) [23], GDC-0152 [24] and LCL161 [25] resemble the amino terminus of processed Smac/Diablo and can interact with a single site within an IAP protein. Bivalent agents, including Birinapant [26], BV6 [27] and SM-164 [28], possess two such moieties. They tend to exhibit higher affinities and potencies than the monovalent drugs, at least *in vitro*, but their size necessitates intravenous administration [29]. IAP antagonists also vary in their reported specificities for XIAP *versus* cIAP1/2. Some, like DEBIO1143/AT406 and Birinapant [23, 26], preferentially target cIAP1 and cIAP2 rather than XIAP. Others, including BV6, LCL161, GDC-0152 and SM-164, have similar affinities for XIAP, cIAP1 and cIAP2 [24, 27, 28, 30].

Early phase clinical trials have revealed that most patients tolerate IAP antagonists, although high doses of at least some can trigger cytokine release syndrome due to their promotion of autocrine TNFα production [31]. As single agents, IAP antagonists triggered complete or partial remissions in a minority of patients with ovarian cancer, colon cancer, melanoma or MALT lymphomas, and stabilized disease in additional patients [29]. More promising data has emerged from studies in which patients were given IAP antagonists with standard anti-cancer therapies. Over a third of poor-risk acute myeloid leukemia patients administered Debio1143 (AT-406) with daunorubicin and cytarabine experienced complete remissions, although half of these subsequently relapsed [32].

Pre-clinical studies revealed that IAP antagonists could also augment the cytotoxicity of other targeted therapies such as chromatin remodeling agents [33-35]. Various IAP antagonists were reported to cooperate with TNF-related apoptosis inducing ligand (TRAIL; Apo2L) to kill carcinoma and leukemia cells *in vitro* and *in vivo* [26, 36-45]. The utility of some of these co-treatments are presently being assessed in clinical trials. In addition to hopefully offering robust anti-cancer efficacy, IAP antagonists lack the mutagenicity associated with DNA damaging chemotherapy [46], so they may spare cancer survivors the risk of developing therapy-related cancers.

In some cell types, exposure to IAP antagonists results in stimulation of non-canonical NF-κB pathways that promote induction of TNFα expression, which stimulates autocrine TNF-R1 signaling of apoptotic and/or necroptotic cell death [47]. IAP antagonists kill these cell types as sole agents. Other cell types fail to produce TNFα following treatment with IAP antagonists. IAP antagonists only kill these cells in the presence of exogenous TNFα produced by other surrounding cells *in vivo*, or provided experimentally *in vitro* [47]. Production of inflammatory cytokines such as TNFα by tumor associated macrophages can enhance the development and progression of various malignancies [48-50]. Recent evidence suggests that osteosarcoma may be a cancer type whose growth and spread is driven by TNFα. TNFα was reportedly required for osteosarcoma progression in mice [51]. Levels of TNFα were elevated in the blood of osteosarcoma patients, particularly those with large tumors [52, 53], and the local concentration at the tumor site would presumably be even higher. The observed overexpression of cIAP1/2 in osteosarcomas [12] probably reflects selective pressure during tumorigenesis for TNFα to stimulate proliferation rather than cell death. Thus the presence of local TNFα probably promotes osteosarcoma expansion and invasion, but could also be exploited therapeutically, if it could cooperate with IAP antagonists to promote tumor cell death.

Most research into the anti-cancer potential of IAP antagonists has focused on carcinomas and hematopoietic malignancies. Very little research has assessed their utility for sarcomas such as osteosarcoma. GDC-0152 was recently published to counter the pro-survival effects of Angiopoietin-like protein 2 on an established human osteosarcoma cell line, SaOS2 [54]. Human osteosarcoma xenografts grew somewhat slower in SCID mice treated with LCL161 than in untreated mice [55], although TNFα levels may be lower in SCID mice than wild type animals [56], so that study may have underestimated the ability of LCL161 to cooperate with host-derived TNFα to kill implanted tumor cells.

In this study we characterized the anti-osteosarcoma activity of a panel of IAP antagonists, using cells derived using two spontaneous osteosarcoma mouse models. Fibroblastic osteosarcomas were isolated from mice in which the p53 and Rb genes were deleted from cells expressing the osteoblast marker *Osterix* [57], whereas osteoblastic osetosarcomas were harvested from animals following lineage-specific deletion of Rb coupled with sh-RNA-mediated p53 downregulation [58].

## RESULTS

### SM-164, GDC-0152 and LCL161 sensitize murine osteosarcoma cells to TNFα

**Figure 1:**
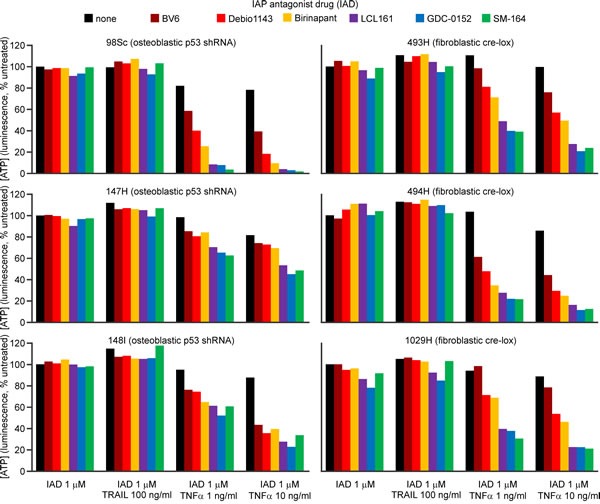
SM-164, GDC-1052 and LCL161 sensitize osteosarcoma cells to TNFα but not TRAIL Cells from the specified primary murine osteosarcomas were incubated for 48 h with the listed IAP antagonist drugs (IAD, colored columns) or media (black columns), together with murine TRAIL or TNFα, or no death ligand. ATP levels were measured with the CellTiterGlo reagent. Analyses of drug interactions and the significance of differences in treatment outcomes are provided in Supplementary Figure 2.

Cells from osteoblastic (98Sc, 147H and 148I) and fibroblastic (493H, 494H and 1029H) primary tumors were incubated with six different IAP antagonists, with or without TRAIL or TNFα. None of the IAP antagonists were toxic to osteosarcoma cells as sole agents or in conjunction with TRAIL (Figure 1, Supp. Figure 1), however LCL161, GDC-0152 and SM-164 cooperated synergistically with TNFα to kill the murine osteosarcoma cells (Figure 1, Supp. Figure 2). Cells from some tumors were more sensitive to the combination treatment than others, but the responsiveness did not correlate with the tumor type (fibroblastic *versus* osteoblastic).

The osteosarcoma cells were only sensitive to IAP antagonists as a co-treatment with TNFα. Use of TNFα as a therapeutic agent has been limited to isolated limb perfusion [59] due to serious toxicities such as hypotension and hepatotoxicity following systemic administration [60]. Therefore, clinical efficacy, particularly for metastatic cancers, would require sufficient endogenous TNFα at the tumor site to cooperate with exogenously administered IAP antagonists. Previous studies documented average TNFα levels in osteosarcoma patients' blood to be 12 [52] and 28 pg/ml [53]. These concentrations were about twice those in the blood of healthy individuals [52, 53], and patients with larger tumors had three-fold higher serum TNFα levels than controls [52]. Although the concentration of TNFα within osteosarcomas has not been reported, presumably the excess TNFα in the blood derives from the tumor site, implying that the intratumoral levels would be much higher.

To define the levels of TNFα required for IAP antagonists to kill murine osteosarcoma cells, we performed dose titration experiments using TNFα and the most potent members of the panel of IAP antagonists: GDC-0152 and SM-164. To focus on physiologically achievable concentrations of the IAP antagonists, we set the upper limit of their concentrations at 10 μM, slightly higher than the GDC-0152 peak plasma concentration (7 μM [24]). Pharmacokinetic data have not been published for SM-164, but the levels of LCL-161 [31] and Birinapant [61] detected in the blood of treated patients resembled those of GDC-0152, so it seems likely that SM-164 may also reach similar levels *in vivo*.

**Figure 2:**
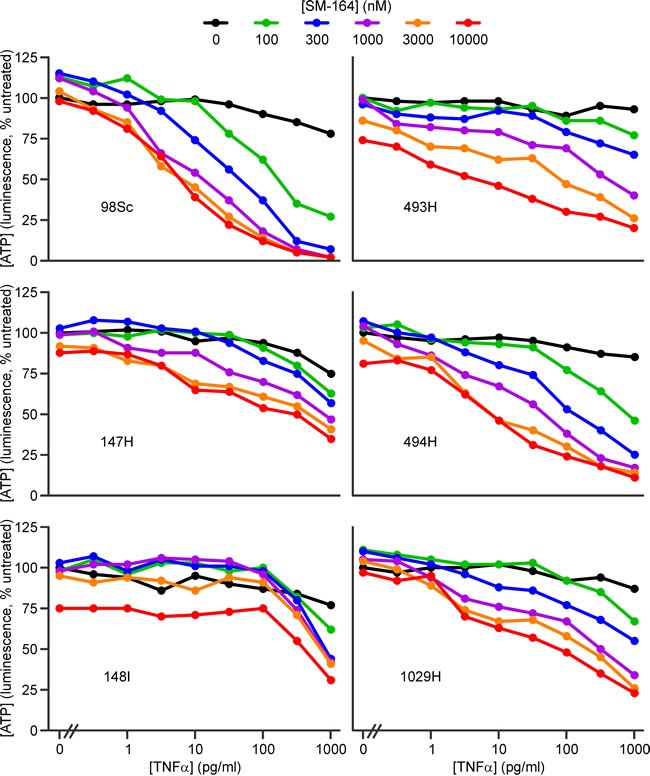
SM-164 cooperates with low concentrations of TNFα to kill cells from some primary murine osteosarcomas Cells from the specified primary murine osteosarcomas were incubated for 48 h with the listed concentrations of SM-164 (colored lines) or media (black columns) together with a range of concentrations of TNFα, or none. ATP levels were measured with the CellTiterGlo reagent. Analyses of drug interactions are provided in Supplementary Figure 3A.

**Figure 3:**
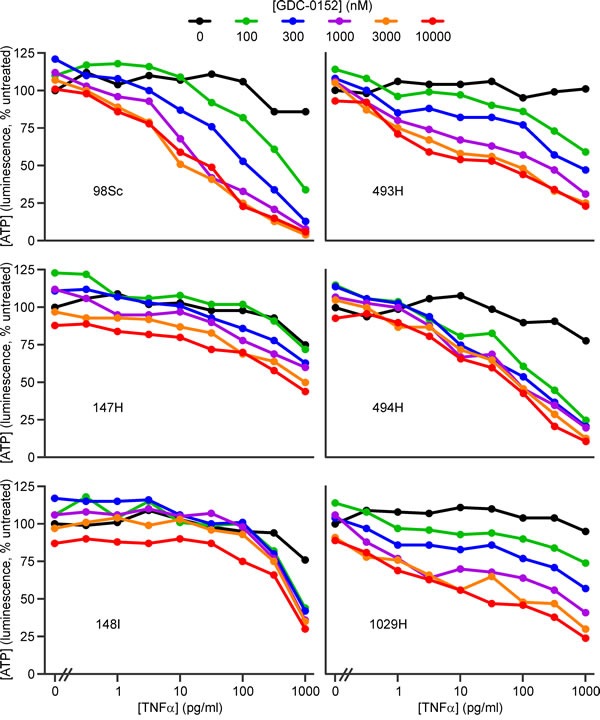
GDC-0152 cooperates with low concentrations of TNFα to kill cells from some primary murine osteosarcomas Cells from the specified primary murine osteosarcomas were incubated for 48 h with the listed concentrations of GDC-0152 (colored lines) or media (black columns) together with a range of concentrations of TNFα, or none. ATP levels were measured with the CellTiterGlo reagent. Analyses of drug interactions are provided in Supplementary Figure 3B.

Both IAP antagonists cooperated with low concentrations of TNFα to kill the osteosarcoma cells (Figure 2, 3, Supp. Figure 3). The extent to which IAP antagonists sensitized cells from the different tumors to TNFα varied. Using ATP levels to monitor survival, cells from four tumors (98Sc, 493H, 494H and 1029H) were killed by levels of SM-164 or GDC-0152 that are likely to be physiologically achievable, coupled with concentrations of TNFα that were similar to or up to 100-fold higher than those detected in the blood of osteosarcoma patients [53]. Cells from tumor 147H exhibited dose-dependent sensitivity to the combined treatment but higher concentrations of both agents were required to kill these cells. Cells from the 148I tumor were also relatively resistant: these cells responded to all concentrations of SM-164 or GDC-0152, but only when co-treated with high concentrations of TNFα. SM-164 seemed to cooperate with TNFα slightly more strongly than GDC-0152 (Figure 2, 3), so the remainder of this study focused on SM-164.

**Figure 4:**
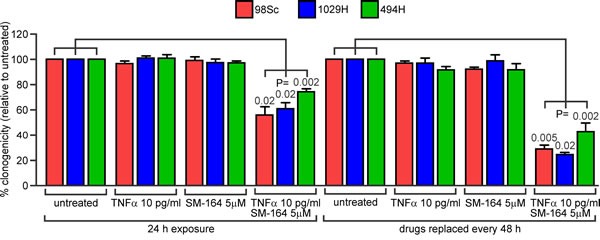
Co-treatment with SM-164 and low concentrations of TNFα reduces the clonogenic survival of osteosarcoma cells 98Sc, 1029H or 494H cells were grown in media containing no drugs or the indicated concentrations of TNFα and/or SM-164,. The drugs were either removed and replaced with normal media after 24h (left side) or replaced every 48 h (right side). The numbers of colonies that arose after these treatments were counted. Mean relative clonogenic survival and SEM are graphed from three independent experiments. Two-sided paired T-tests were used to calculate the probability that the observed differences between untreated cells and cells treated with TNFα plus SM-164 were due to chance. Analyses of the significance of differences between results from single and combined treatments, and raw data, are provided in Supplementary Tables 1 and 2.

To verify that the decrease in ATP levels observed following combined treatment with SM-164 and TNFα reflected abolition of clonogenic survival, we exposed primary tumor cells from three mice to a concentration of SM-164 likely to resemble the peak plasma level (5 μM) together with a concentration of TNFα slightly lower than that reported in the blood of osteosarcoma patients (10 pg/ml). Clonogenic survival was monitored after a 24 h exposure or after continuous exposure (replacing media containing or lacking the drugs every 48 h). Combined treatment for 24 h reduced the cells' clonogenicity by 25-40%, and extended exposure abolished the clonogenicity of the majority of the cells (Figure 4, Supplementary Tables 1, 2).

### Doxorubicin cooperates with IAP antagonists plus TNFα to kill osteosarcoma cells

**Figure 5:**
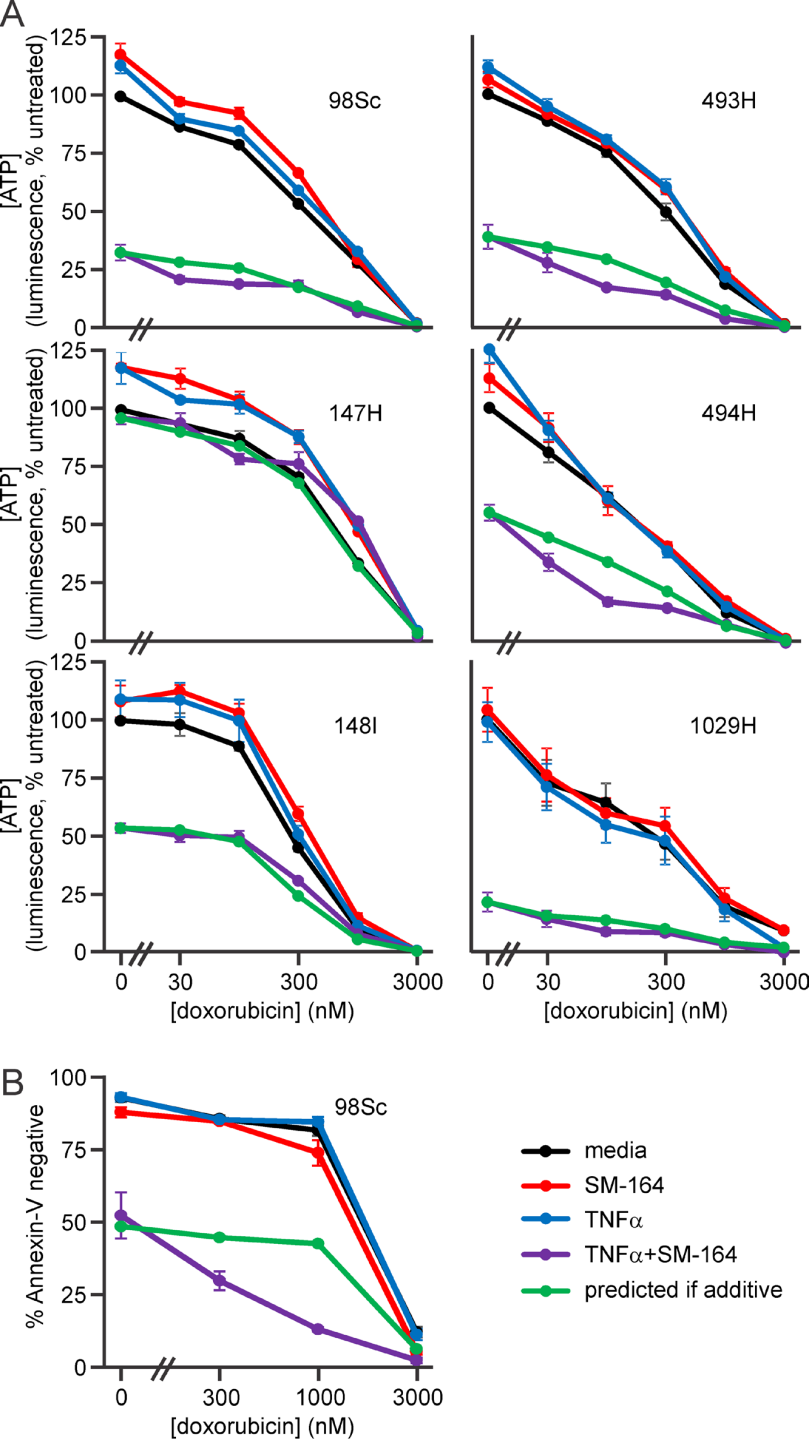
Doxorubicin cooperates with SM-164 and TNFα co-treatment to kill primary murine osteosarcoma cells **A.** Osteosarcoma cells from the specified murine tumors were incubated in media containing no drugs, 1 μM SM-164 and/or 1 ng/ml TNFα with the indicated concentrations of doxorubicin for 48 h. ATP levels in the cells were then measured using the CellTiterGlo kit. **B.** 98Sc cells were incubated in media containing no drugs, 10 μM SM-164 and/or 1 ng/ml TNFα with the indicated concentrations of doxorubicin for 48 h, stained with Annexin-V-FITC to identify dead and dying cells, then analyzed by flow cytometry. (A, B) The green line shows the expected outcomes based on an additive interaction between SM-164/TNFα and doxorubicin, calculated using a fractional product approach. Means and SEM from three independent experiments are shown. Analyses of drug interactions are provided in Supplementary Figure 4.

Doxorubicin is a crucial component of multi-agent chemotherapy regimens for osteosarcoma patients [4]. We therefore explored whether SM-164 and TNFα could enhance the anti-osteosarcoma activity of doxorubicin. Cells from each of the tumors were incubated with no drugs, SM-164, TNFα or both, coupled with a range of doxorubicin concentrations up to that detected in the blood of treated cancer patients [62]. Increasing concentrations of doxorubicin enhanced the lethality mediated by co-treatment with SM-164 plus TNFα (Figure 5). The magnitude of this cooperation was similar to that predicted for an additive interaction (Figure 5, Supp Figure 4).

### Sensitization of osteosarcoma cells to TNFα by IAP antagonists requires RIPK1

**Figure 6:**
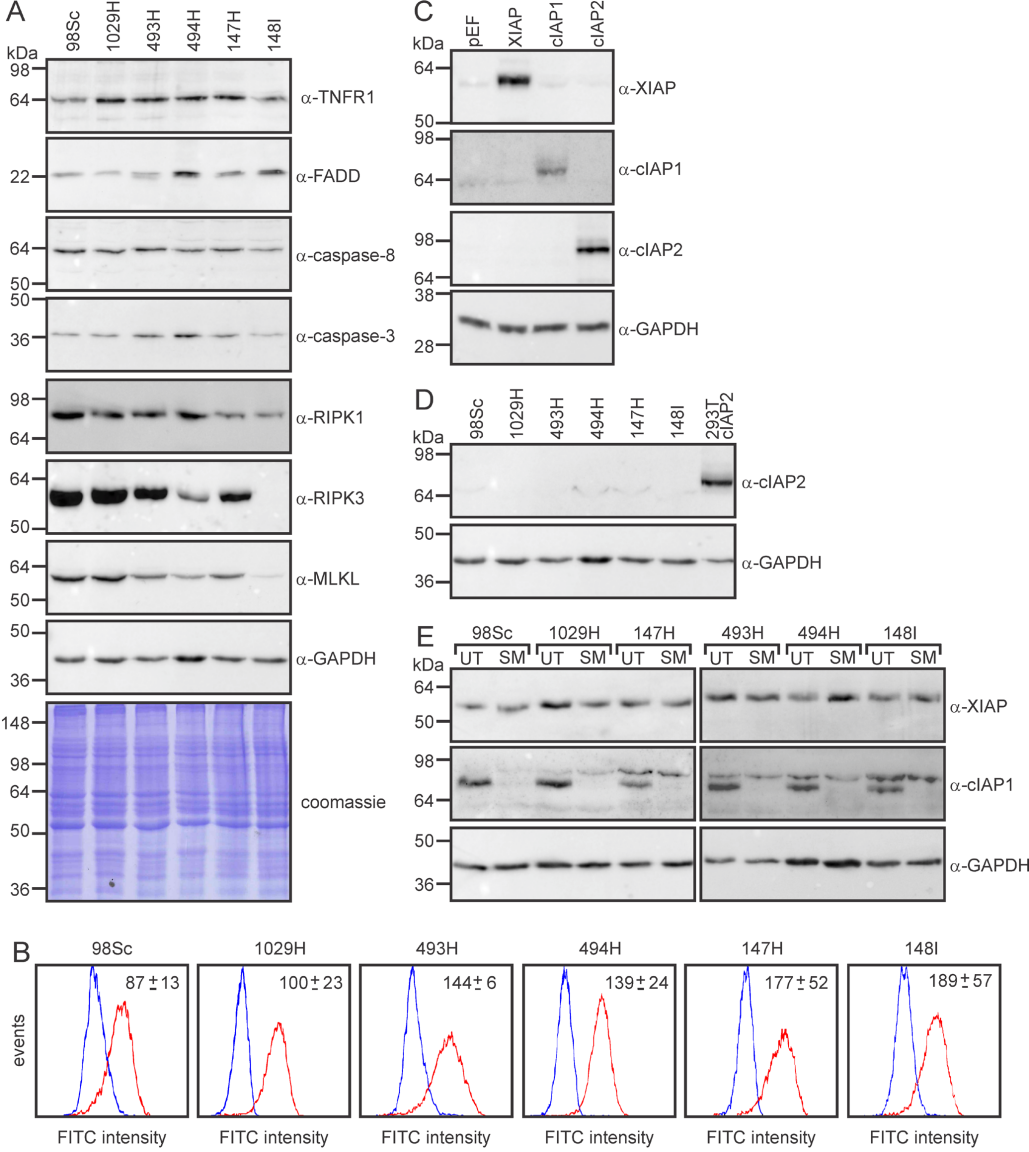
RIPK1 and RIPK3 levels vary between primary murine osteosarcomas **A.** Cells from the specified tumors were lysed and subjected to SDS-PAGE, then immunoblotted with the indicated antibodies or subjected to Coomassie staining. **B.** Unpermeabilized cells were stained with anti-TNFR1 antibody (red lines) or anti-GFP (as a control; blue lines) followed by FITC-conjugated anti-rabbit, then analyzed by flow cytometry. The average specific mean fluorescence intensities (anti-TNFR1 minus anti-GFP) are stated (+/− SEM, from three experiments). **C.** To validate the specificity of the IAP family member antibodies, 293T cells were transfected with plasmids encoding the specified proteins or empty vector (pEF), then lysates were immunoblotted with the listed antibodies. **D.** Lysates from the osteosarcoma cells and 293T cells transfected with a cIAP2 expression plasmid were immunoblotted with an antibody recognizing cIAP2. **E.** Cells were incubated in media (UT) or 10 μM SM-164 (SM) for 45 min then lysed and subjected to immunoblotting with the listed antibodies.

To explore the molecular basis for the heterogeneity in responses we observed between cells derived from different tumors, particularly the relative resistance of cells from tumors 147H and 148I, we surveyed expression of proteins previously implicated in cell death induced by IAP antagonists. Antagonism of IAP proteins can sensitize cells to TNFα-mediated apoptosis, which requires the “ripoptosome” components RIPK1, caspase-8 and FADD, and/or necroptosis, which typically involves the “necrosome” constituents RIPK1, RIPK3 and MLKL. The levels of surface and total expression of the receptor TNF-R1, and intracellular levels of FADD and caspases-8 and -3 were no lower in the resistant cells than the sensitive ones (Figure 6A, 6B). Cells from each of the tumors expressed similar amounts of XIAP and cIAP1, and none expressed detectable levels of cIAP2 (Figure 6C-6E). SM-164 treatment stimulated equally dramatic degradation of cIAP1 in cells from each of the osteosarcomas (Figure 6E). Interestingly, the two more resistant cell lines, 147H and 148I, expressed less RIPK1 than cells from the other tumors (Figure 6A). Cells from the 148I tumor also lacked any detectable RIPK3 expression and bore only low levels of MLKL (Figure 6A).

**Figure 7:**
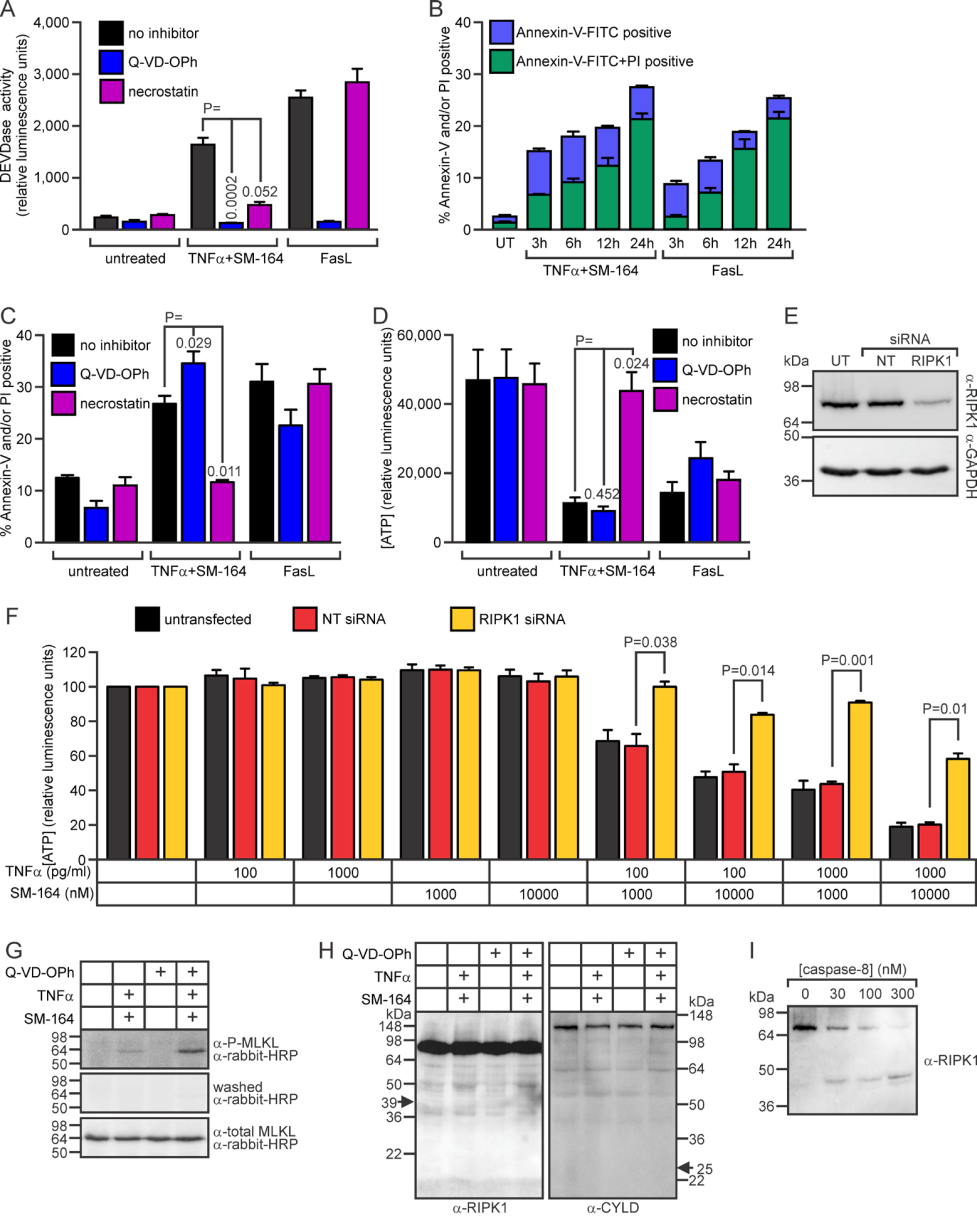
Co-treatment with SM-164 plus TNFα stimulates RIPK1-dependent apoptosis of 98Sc osteosarcoma cells **A.** 98Sc cells were pre-treated with no inhibitor, Q-VD-OPh (to inhibit caspases) or necrostatin (to inhibit RIPK1) for 1 h, then additional media was added containing either no drugs, 1 ng/ml TNFα plus 1 μM SM-164, or 100 ng/ml FasL (all final concentrations). After 6 h, DEVDase (caspase) activity was measured. **B.** 98Sc cells were incubated with 1 ng/ml TNFα plus 1 μM SM-164 or 100 ng/ml FasL for the designated periods of time. The cells were stained with propidium iodide (PI) and Annexin-V-FITC to identify dead and dying cells and analyzed by flow cytometry. **C.** 98Sc cells were treated as outlined for panel A. After 6 h, cell death was monitored flow cytometrically after staining with Annexin-V-FITC and propidium iodide. **D.** 98Sc cells were treated as outlined for panel A. ATP levels, reflecting the proportion of surviving and metabolically active cells, were measured after 24 h. 494H cells were transfected with a non-targeting siRNA (NT) or an siRNA targeting RIPK1, or left untransfected (UT). Cells were harvested for anti-RIPK1 immunoblotting **E.** and for seeding into plates containing the specified concentrations of drugs. After 48 h, ATP levels were measured **F. G.**-**I.** 98Sc cells were lysed following 6 h incubation in media or 1 ng/ml TNFα plus 10 μM SM-164, with or without 1 h pre-treatment with Q-VD-OPh. (G) Lysates were subjected to SDS-PAGE and immunoblotting with an antibody recognizing phospho-MLKL. The membrane was washed and probed with secondary antibody (to check for residual signal) before being probed with an antibody recognizing phosphorylated and unphosphorylated MLKL (and exposed for the same time as following probing only with secondary antibody). (H) Lysates were immunoblotted to detect RIPK1 or CYLD. Arrows indicate the expected migration of predicted cleavage products. (I) Lysate from untreated cells was incubated with recombinant caspase-8 at 37°C for 30 min before SDS-PAGE and immunoblotting to detect RIPK1. (A-D, F) Means and SEM are shown from three independent experiments. (A, C, D, F) P values are shown from two sided, paired t-tests, comparing the responses to the indicated treatments.

Treating cells from the 98Sc tumor with SM-164 and TNFα provoked DEVDase activity (Figure 7A), which has been shown to predominantly reflect levels of active caspases-3, -7 and possibly -8 [63, 64]. The combined treatment also led to externalization of phosphatidyl serine, prior to disruption of the plasma membrane, similar to the classical caspase-dependent apoptosis provoked by exposure to Fas ligand (Figure 7B). The lethality and DEVDase activity stimulated by SM-164/TNFα co-treatment were efficiently inhibited by necrostatin (Figure 7A, 7C, 7D), and downregulation of RIPK1 in 494H cells reduced the loss of ATP in these cells following co-treatment with SM-164 and TNFα (Figure 7E, 7F). These data imply that SM-164/TNFα-induced death involved RIPK1-dependent activation of caspase-8 and executioner caspases [65]. Pan-caspase inhibition by Q-VD-OPh [66] suppressed the DEVDase activity associated with SM-164/TNFα treatment (Figure 7A), but the caspase inhibitor slightly augmented rather than suppressed the death following the co-treatment (Figure 7C, 7D). MLKL phosphorylation was markedly induced in cells treated with Q-VD-OPh before exposure to SM-164/TNFα (Figure 7G), suggesting that necroptotic death was triggered by TNFα when IAPs and caspase were inhibited. Caspase-8 has been shown to suppress necroptosis [67, 68], so presumably in this context Q-VD-OPh indirectly stimulated necroptosis by preventing the proteolysis of one or more pro-necroptotic substrates by caspase-8 [22]. RIPK1 has been proposed to be a key substrate whose cleavage by caspase-8 suppresses necroptosis [69-72], although evidence that RIPK1 is a poor caspase-8 substrate [73, 74] argues against this model. CYLD, an enzyme that de-ubiquitinates RIPK1 [75], was also reported to be a crucial substrate whose proteolysis by caspase-8 inhibits necroptosis [76]. We failed to detect cleavage of either of these proteins in cells treated with SM-164/TNFα, with or without prior Q-VD-OPh treatment (Figure 7H). We therefore speculate that, in these osteosarcoma cells, TNFα/SM-164-induced necroptosis triggered by caspase inhibition resulted from suppression of caspase-8-mediated cleavage of an as-yet unidentified substrate.

### SM-164 sensitizes primary and metastatic osteosarcoma cells to TNFα

**Figure 8:**
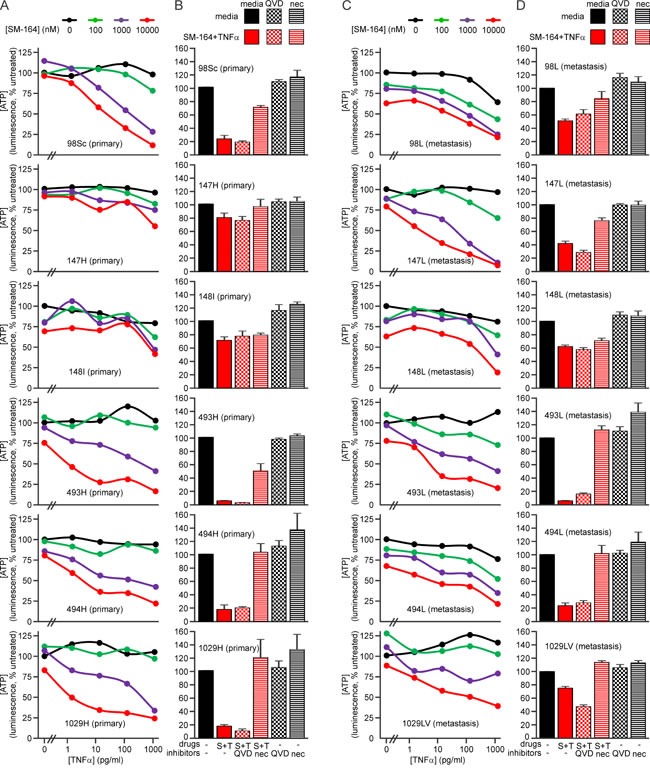
SM-164 sensitizes metastatic murine osteosarcoma cells to TNFα **A.**, **C.** Cells from primary (A) or metastatic (C) tumors were incubated in the media containing no drugs or a range of concentrations of SM-164 and/or TNFα for 48 h, then ATP levels were measured. **B.**, **D.** Cells from the specified primary (B) or metastatic (D) tumors were pre-treated with no inhibitor, 10 μM of Q-VD-OPh (to inhibit caspases) or 10 μM necrostatin (to inhibit RIPK1) for 1 h, then additional media was added containing either no drugs or SM-164 plus TNFα. Ten micromolar SM-164 was used for cells from all tumors. TNFα was used at 1 ng/ml for the more resistant cells (147H and 148I) and 100 pg/ml for the remainder. ATP levels, reflecting the proportion of surviving and metabolically active cells were measured after 48 h. Means and SEM are shown from three independent experiments.

**Figure 9:**
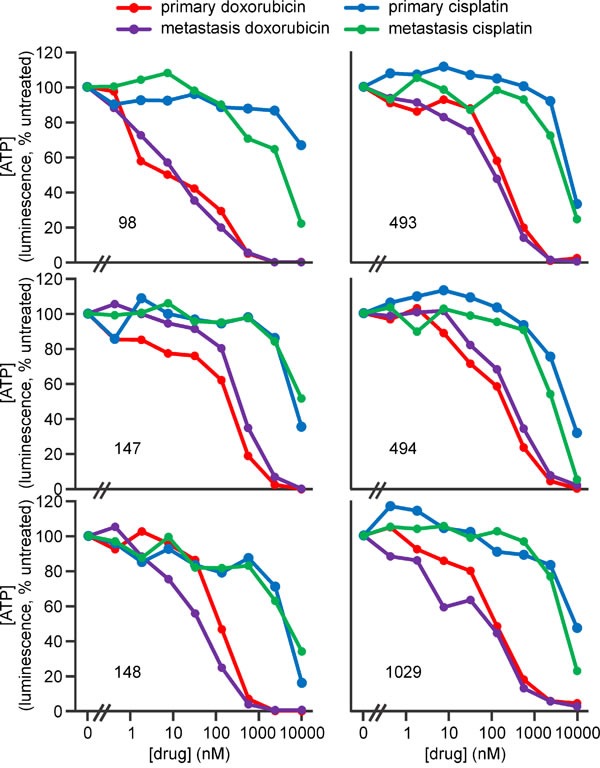
Doxorubicin and cisplatin kill murine primary and metastatic osteosarcoma cells Osteosarcoma cells from murine early passage cell lines derived from primary or metastatic tumors from the numbered mice were incubated in the media containing no drugs or the indicated concentrations of doxorubicin or cisplatin for 48 h, then ATP levels were measured. Analyses of the significance of differences in sensitivity of cells from primary and metastatic tumors are provided in Supplementary Figure 5.

Metastatic osteosarcomas are particularly unresponsive to current therapies. One of the strengths of the genetically engineered murine osteosarcoma models used in this study is their ability to recapitulate the typical metastatic pattern of human osteosarcoma; primarily metastasizing to lung and less commonly to other sites including liver [57, 58, 77]. Most of the mice whose primary tumors were analyzed in the preceding experiments developed lung metastases (denoted by an “L” suffix) and one had a secondary tumor in its liver (#1029LV). We examined the sensitivity of cells from these secondary osteosarcomas to SM-164 and TNFα in parallel with cells from the matching primary tumors. Cells from all of the metastatic osteosarcomas were sensitive to the combination treatment (Figure 8, Supplementary Table 3). Interestingly, however, cells from the lung metastasis in mouse #147 were more sensitive than cells from that animal's primary tumor, whereas cells from the metastatic tumors from mice #98 and #1029 were somewhat less sensitive than cells from their primary tumors (Figure 8). These data suggest that TNFα signaling pathways changed during metastasis in these animals. However, osteosarcomas are highly penetrant in these genetically engineered mice, so it is also possible that some of the metastatic tumors arose independently, from a distinct primary cancer from that which we resected and analyzed. In contrast, cells from primary and metastatic tumors from each mouse generally exhibited similar sensitivity to doxorubicin and cisplatin, as measured by ATP levels (Figure 9, Supp Figure 5), although cisplatin exposure provoked a more substantial drop in ATP levels in cells from the metastatic tumor from mouse #98 than to those from its primary tumor (Figure 9).

**Figure 10:**
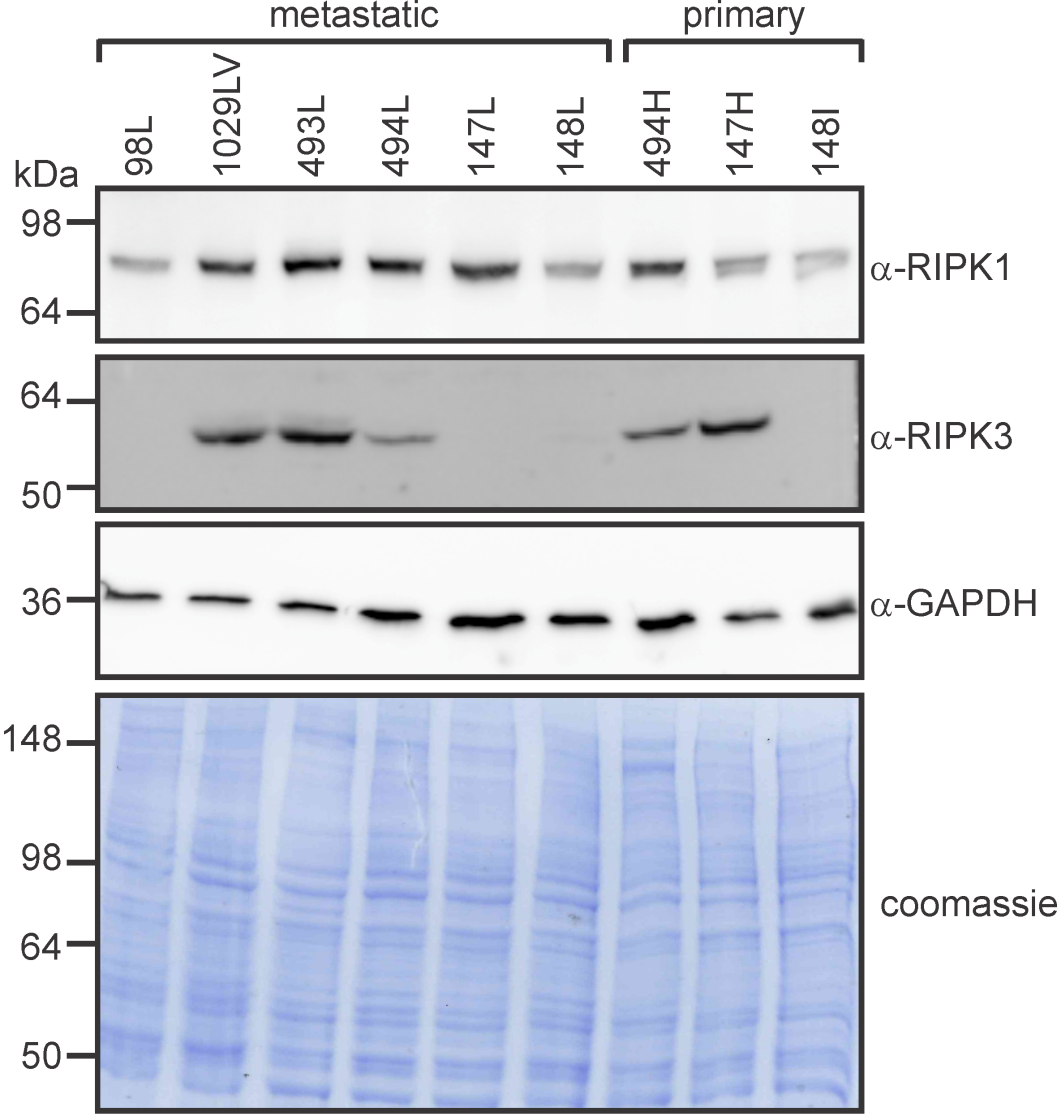
RIPK1 and RIPK3 levels vary between primary and metastatic murine osteosarcomas Cells from the specified tumors were lysed and subjected to SDS-PAGE, then immunoblotted with the indicated antibodies or subjected to Coomassie staining.

Cells from the lung metastases 98L and 148L, which were relatively insensitive to SM-164/TNFα-induced death (Figure 8), expressed comparatively little RIPK1 (Figure 10). Interestingly, RIPK3 levels varied considerably in cells from the metastatic osteosarcomas: 1029LV and 493L expressed robust levels, 494L expressed less, and none was detected in lysates of cells from the metastatic tumors 98L, 147L or 148L (Figure 10). The RIPK3 levels did not correlate with sensitivity, however. Cells from the metastatic tumor 147L lacked RIPK3 expression, yet SM-164 sensitized these cells to TNFα as efficiently as cells from the RIPK3-expressing tumors 493L and 1029LV. Necrostatin completely prevented or substantially reduced SM-164/TNFα-mediated death of cells from most of the tumors (Figure 8). Although necrostatin completely blocked the lethality to 98Sc cells of 1 μM SM-164 plus 1 ng/ml of TNFα (Figure 7), it was less effective at suppressing death provoked by co-treatment of these highly responsive cells with 10 μM SM-164 coupled with 100 pg/ml of TNFα (Figure 8). Necrostatin was also less active on cells expressing low levels of RIPK1 (from the primary tumor 148I and metastases 98L, 147L and 148L). Caspase inhibition by Q-VD-OPh failed to suppress SM-164/TNFα-induced death in cells from any tumors.

### Unlike doxorubicin, SM-164 does not provoke mutations in surviving cells

**Figure 11:**
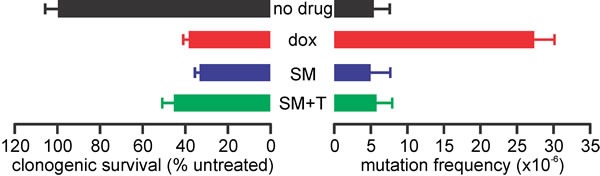
SM-164 does not induce mutations at the HPRT locus of TK6 cells TK6 cells were incubated for 24 h with no drug, 5 nM doxorubicin (dox), 15 μM SM-164 (SM) or 15 μM SM-164 plus 5 pg/ml TNFα (SM+T). Clonogenic survival was determined (left bars) or the cells were seeded in 96-well plates containing or lacking 6-thioguanine to determine the HPRT mutation frequency (right bars). Means and SEM from three independent experiments are shown.

An emerging challenge in cancer treatment is minimizing the risk of survivors acquiring subsequent malignant neoplasms due to their exposure to DNA damaging chemotherapy or radiotherapy [78]. We previously found that the IAP antagonists LCL161 and DEBIO1143 (AT-406) were non-mutagenic [46], implying that they would lack the oncogenic potential of current anti-cancer drugs. To determine whether SM-164 also lacks mutagenic activity, we compared the ability of equivalently lethal concentrations of doxorubicin and SM-164 (alone or with a low concentration of TNFα) to provoke loss-of-function mutations in the HPRT locus of TK6 lymphoid cells. This assay capitalizes on the ability of active HPRT enzymes to convert the purine analog 6-thioguanine into a toxin, hence the clonogenicity of drug-treated TK6 cells grown in the presence of 6-thioguanine provides a measure of the drug's ability to induce mutations in the HPRT gene [79]. A concentration of doxorubicin that abolished the clonogenicity of around 40% of the TK6 cells provoked frequent emergence of 6-thioguanine resistant clones (implying HPRT mutations) (Figure 11). In contrast, a concentration of SM-164 that had a similar impact on clonogenicity failed to yield any more 6-thioguanine-resistant clones than no treatment. Addition of a physiologically-relevant concentration of TNFα did not affect clonogenicity or mutagenicity (Figure 11).

## DISCUSSION

IAP antagonists cooperated with low doses of TNFα to kill cells from most primary and metastatic murine osteosarcomas, suggesting that members of this class of drugs may be therapeutically useful anti-osteosarcoma agents. The cooperation we observed between SM-164/TNFα and doxorubicin further suggests that IAP antagonists may augment the efficacy of currently used chemotherapy regimens, and/or could permit lower (safer) doses of chemotherapy drugs to be employed without compromising anti-tumor responses.

It was intriguing that the murine osteosarcoma cells survived combined treatment with TRAIL and IAP antagonists, despite expressing the death-promoting murine TRAIL receptor (Supp Figure 1). IAP antagonism was previously published to sensitize cells from breast, prostate, colon, urothelial, cervical, nasopharyngeal, bladder and pancreatic cancers, leukemias, neuroblastomas and gliomas to killing by TRAIL [26, 36, 38, 39, 43-45, 80-85], so the resistance of osteosarcoma cells to this combined treatment is unusual. Further work will be required to determine the molecular basis for this resistance. Possible mechanisms could include expression of membrane-bound decoy receptors mDcTRAIL-R1/2 [86] or the soluble decoy receptor osteoprotegerin (OPG), which is produced by osteoblasts [87] and osteosarcomas [88] and has been previously shown to protect osteosarcoma [89], breast cancer [90] and myeloma [91] cells from TRAIL-induced death.

The IAP antagonists that cooperated most strongly with TNFα to kill osteosarcoma cells efficiently targeted XIAP in addition to cIAP1 and 2. Treatment of the sensitive osteosarcoma cells with SM-164 plus TNFα provoked rapid DEVDase activity and exposure of phosphatidyl serine, followed by subsequent plasma membrane disruption. The responsive cells could be protected by pre-treatment with necrostatin, and cells from some of the sensitive osteosarcomas expressed little or no RIPK3. Together these observations suggest that, for TNFα to kill osteosarcoma cells, RIPK1 is required and the activities of cIAP1 and XIAP must be diminished. We infer that, in osteosarcoma cells, IAP antagonist-mediated degradation of cIAP1 enables formation of the ripoptosome and activation of caspase-8, which can stimulate caspase-3/7 activity if the IAP antagonist permits this by relieving XIAP-mediated inhibition. Presumably Birinapant and DEBIO1143/AT-406 were less effective because they target XIAP less efficiently than SM-164, GDC-0152 and LCL161. BV6 appears to represent an exception to this rule: its affinity for XIAP was reportedly only around three-fold lower than for cIAP1 [27] yet it was the least potent IAP antagonist of those tested in this study. It is possible that this surprisingly marginal impact of BV6 plus TNFα reflects less efficient uptake of BV6 than the other drugs, rather than differences in their biochemical activities.

RIPK1 expression levels predicted sensitivity to IAP antagonist/TNFα-induced death in cells from the different tumors. Necrostatin reduced or abolished the lethality triggered by treatment of osteosarcoma cells with IAP antagonists plus TNFα, implying that RIPK1 is required for this cell death. In contrast, caspase inhibition (by Q-VD-OPh) failed to protect these cells from IAP antagonist/TNFα-mediated death. Phosphorylation of MLKL in 98Sc cells under these conditions suggests that inhibition of caspases (probably caspase-8) switched signaling from ripoptotic to necroptotic cell death, consistent with previous reports [67, 68]. We were however surprised to note that caspase inhibition was ineffectual even in cells lacking RIPK3, the kinase proposed to be responsible for MLKL phosphorylation [92]. Cells from one primary and three secondary tumors lacked detectable RIPK3 expression, however this did not prevent their killing by treatment with IAP antagonists and TNFα. Cells from the 147L lung metastasis, which expressed robust levels of RIPK1 but no RIPK3, were as sensitive as cells from other tumors that expressed similar levels of RIPK1 and high levels of RIPK3. Q-VD-OPh slightly enhanced the sensitivity of some cells to SM-164/TNFα, and had negligible effects on others. In contrast, necrostatin markedly reduced the sensitivity of cells from all tumors. Thus, our data suggest that although IAP antagonist/TNFα treatment of osteosarcoma cells normally leads to ripoptosome-mediated apoptosis, suppression of caspase activity in osteosarcoma cells treated with these agents provokes cell death that is primarily RIPK1-dependent but does not necessarily require RIPK3. A similar observation was recently reported in murine fibrosarcoma cells [93]. Necrostatin failed to protect primary or metastatic cells from mouse #148 from death caused by treatment with high doses of SM-164 and TNFα. These cells lacked RIPK3 and expressed low levels of RIPK1, and were relatively unresponsive to IAP antagonists plus TNFα. These data suggest that high doses of SM-164 plus TNFα can reduce the survival of cells from this tumor through pathways that are independent of caspases, RIPK1 and RIPK3, but because high concentrations of drugs were required to stimulate this death *in vitro*, such alternative pathways may not occur *in vivo*.

The ability of necrostatin to protect sensitive osteosarcoma cells from death triggered by co-treatment with IAP antagonists plus TNFα imply that RIPK1 is an important determinant of sensitivity. Consistent with this, the less sensitive osteosarcoma cell lines expressed lower levels of RIPK1. If the results of this study translate to human osteosarcomas, the levels of TNFα and RIPK1 in patients' tumors may predict their responses to treatment with pan-specific IAP antagonists. Because IAP antagonists including SM-164 are non-mutagenic, we expect that they would be less likely than DNA-damaging chemotherapy drugs to stimulate therapy-induced cancers in cured osteosarcoma patients.

## MATERIALS AND METHODS

### Reagents

Drugs used in this study were recombinant murine sTRAIL/Apo2L (Peprotech; NJ, USA), murine TNFα (Peprotech), Debio1143/AT-406 (Selleck Chemicals; Texas, USA), LCL161 (Selleck Chemicals), SM-164 (ApexBio; Texas, USA), Birinapant (ApexBio), BV6 (ApexBio), GDC-0512 (Selleck), doxorubicin (Sigma-Aldrich; MO, USA) and cisplatin (Sigma-Aldrich). The following antibodies were used: anti-cIAP1 (Enzo Life sciences; NY, USA), anti-cIAP2 (R&D systems; MN, USA), anti-XIAP (MBL International; MA, USA), anti-FADD (Abcam; Cambridge, UK), anti-TNFR1 (Abcam), anti-TRAIL-R2 (R&D systems), anti-caspase-3 (BD Biosciences; NJ, USA), anti-caspase-8 (Abcam), anti-RIPK1 (BD Biosciences), anti-RIPK3 (ProSci; CA, USA), anti-MLKL1 (Abcam), anti-phosphoS345-MLKL (Abcam), anti-CYLD (Thermo Fisher Scientific; Massachusetts, USA), anti-crmA (Santa Cruz Biotechnology; Texas, USA), rabbit anti-GFP (polyclonal in-house antibody), goat anti-rabbit FITC (Merck Millipore; MA, USA), rabbit anti-goat-FITC (Thermo Fisher Scientific), mouse anti-GAPDH (Merck Millipore), donkey anti-rabbit HRP conjugated antibody (GE Healthcare Life Sciences; NJ, USA), goat anti-rat HRP conjugated antibody (GE Healthcare Life Sciences), and rabbit anti-mouse-HRP conjugated antibody (Sigma).

### Animals and cells

Spontaneous fibroblastic primary and metastatic osteosarcomas were obtained from *Osx*-Cre p53^fl/fl^ pRb^fl/fl^ mice [57]. Osteoblastic osteosarcomas were harvested from *Osx*-Cre TRE-p53.1224 pRb^fl/fl^ mice [58]. Cells were isolated from the tumors by dissociation with a scalpel and cultured from these tumors as described previously [94], and used within 14 *ex vivo* passages. TK6 cells [95] were obtained from ATCC (Manassas, Virginia, USA) and were grown in RPMI (Invitrogen; CA, USA) containing 10 % heat inactivated FBS. L929 cells (a kind gift from Wendy Cook and David Vaux), HEK-293T cells and LN18 cells and were cultured in Dulbecco's modified Eagle medium with high glucose (Invitrogen) supplemented with 10 % heat inactivated FBS. All cells were grown at 37°C in air supplemented with 5 % CO_2_.

### Cell death and caspase assays

For clonogenicity assays, one hundred thousand cells were plated per well of a 24-well plate and incubated with drugs or normal media for the specified times, then washed once with phosphate buffered saline (PBS; Astral Scientific; Tarren Point, NSW, Australia) and plated at various densities in 6-well plates. After seven days (494H) or ten days (98Sc and 1029H) or fourteen days (147H and 148I), cells were stained with methylene blue (Sigma-Aldrich; 1.25 g/l in 50 % methanol) for 5 min, washed twice with water, and then the numbers of colonies were counted.

For acute cell death experiments, cells incubated with drugs or media were harvested, washed once with PBS, then resuspended in binding buffer (10 mM Hepes, 140 mM NaCl, 2.5 mM CaCl2; pH 7.4) containing 1:250 of Annexin-V-FITC (Abcam). After incubating the cells at 4°C for 15 min, an equal amount of binding buffer containing 2 μg/ml propidium iodide (Sigma-Aldrich) was added before analysis. Cells were analyzed by flow cytometry for propidium iodide and/or Annexin-V-FITC positive cells using FACS Canto II (BD Biosciences). In some experiments cells were pre-treated for 1 h with 10 μM of the pan-caspase inhibitor Q-VD-OPh (R&D systems) or the RIPK1 inhibitor necrostatin-1 (Sigma-Aldrich).

The amount of ATP or DEVDase activity in cells upon treatment were determined using CellTiter-Glo 2.0 or Caspase-Glo 3/7 assay kits respectively (Promega; WI, USA). Two thousand cells per well were plated in 96-well white plates containing desired drugs or media to a final volume of 75 μl, and incubated for specified time. In some experiments cells were pre-treated for 1 h with 10 μM of Q-VD-OPh (R&D systems) or necrostatin-1 (Sigma-Aldrich). After treatment, 75 μl of CellTiter-Glo or Caspase-Glo 3/7 solution was mixed into each well. The plates were incubated at room temperature for 10 min (CellTitre-Glo) or 30 min (Caspase-Glo3/7) then luminescence was recorded using a Spectromax M5 (Molecular Devices; CA, USA).

### Transfections

HEK-293T cells were seeded into 6-well plates (3 × 10^5^ cells per well) for transfection on the following day. Cells were transfected using Lipofectamine 2000 (Thermo Fisher Scientific) with previously published plasmids [96], and then harvested 24 h post-transfection for immunoblotting. For siRNA transfections, 494H cells were seeded into 6-well plate (1.6 × 10^4^ cells per well) in antibiotic free media. The following day cells in each well were transfected with 50 nM of On-Targetplus non-targeting siRNA #2 (Thermo Fisher Scientific) or SMART pool: On-Targetplus mouse RIPK1 siRNA (Thermo Fisher Scientific) using 2 μl of DharmaFECT-2 transfection reagent (Thermo Fisher Scientific). Cells were incubated with transfection mixture for 8 h, before replacing media. Transfected cells were cultured for 48 h, and then harvested for immunoblotting and cell death assays.

### Flow cytometry

Expression of TNFR1 or TRAIL-R2 on the surface of murine osteosarcoma cells was determined by flow cytometry. Cells were harvested and fixed in 4 % paraformaldehyde (Sigma-Aldrich) for 15 min at 4°C. Fixed cells were washed with PBS containing 4 % FBS (PFS), then resuspended in 100 μl of PFS containing anti-TNFR1 antibody (or equivalent concentration of anti-GFP antibody as an isotype control), or anti-TRAIL-R2 antibody (or equivalent concentration of anti-crmA as an isotype control). Samples were incubated, rotating, at 4°C overnight, followed by washing twice with PFS and incubating with 100 μl of PFS containing FITC-conjugated secondary antibody for 1 h at room temperature, rotating. Samples were washed twice with PFS and FITC positive cell populations were determined using a FACS Canto II (BD Biosciences).

### Cell lysis, electrophoresis and immunoblotting

Cell samples were lysed using RIPA lysis buffer (150 mM sodium chloride, 1.0 % Triton X-100, 0.5 % sodium deoxycholate, 0.1 % SDS, 50 mM Tris, pH 8.0) supplemented with protease inhibitor cocktail (Roche; Basel, Switzerland), then forced 10 times through a 23-gauge needle to shear the DNA. The lysates were cleared by centrifuging for 15 min at 16,100 g at 4°C. Total protein was determined using the bicinchoninic acid (BCA) method (Micro BCA Protein assay kit, Thermo Fisher Scientific; IL, USA). Thirty to forty micrograms of lysates were loaded on Tris-glycine gels and the proteins were separated by sodium dodecyl sulfate-polyacrylamide gel electrophoresis. The proteins were then either stained with coomassie stain followed by de-staining, or transferred onto Hybond PVDF 0.22 μm membrane (Millenium Science; Victoria Australia), blocked with 1% blocking reagent (Roche) in phosphate-buffered saline (PBS), and probed with primary antibodies in 1% blocking reagent (Roche) in PBS with 0.1% Tween-20 (Sigma-Aldrich). Horseradish peroxidase (HRP)-conjugated secondary antibodies were detected using SuperSignal West Dura extended duration substrate (Thermo Fisher Scientific).

### Mutagenesis

HPRT mutagenesis was performed as previously described [42] except that 2×10^4^ cells were seeded per well in 96 well plates the presence of 30 μM 6-thioguanine.

## SUPPLEMENTARY MATERIAL FIGURES AND TABLES


