# Supplementary material for: IAP antagonists sensitize murine osteosarcoma cells to killing by TNFα
Source: Oncotarget. 2016 Apr 25;7(23):33866–86. doi: 10.18632/oncotarget.8980 (PMC5085125; doi:10.18632/oncotarget.8980)
Supplement: Supplementary file 1 [file oncotarget-07-33866-s001.pdf]

IAP antagonists sensitize murine osteosarcoma cells to killing by TNFα

Supplementary Material

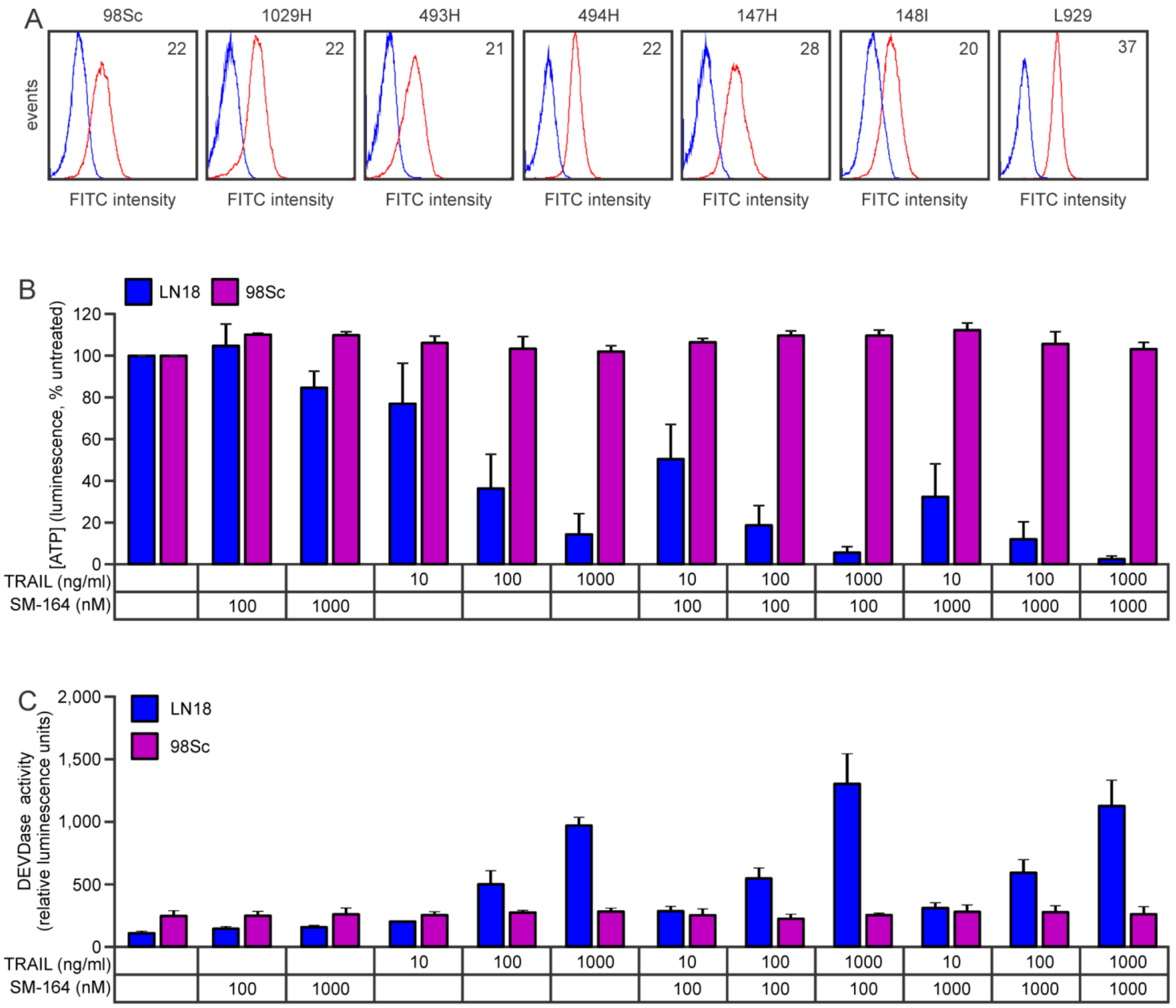

Supplementary Figure 1: Osteosarcoma cells express TRAIL-R2 but are not sensitized to TRAIL-induced death by SM-164. (A) Unpermeabilized osteosarcoma or L929 murine fibrosarcoma cells were stained with anti-TRAIL-R2 antibody (red lines) or anti-crmA (as a control; blue lines) followed by FITC-conjugated anti-goat, then analyzed by flow cytometry. The specific fluorescence intensities (anti-TRAIL-R2 minus anti-crmA) are stated. (B-C) 98Sc or LN18 (human glioma) cells were treated with no drugs, murine TRAIL and/or SM-164 at the indicated concentrations. (B) ATP levels, reflecting the proportion of surviving and metabolically active cells, were measured after 48 h. (C) DEVDase (caspase) activity was measured after 6 h. (B, C) Means and SEM are shown from three independent experiments.

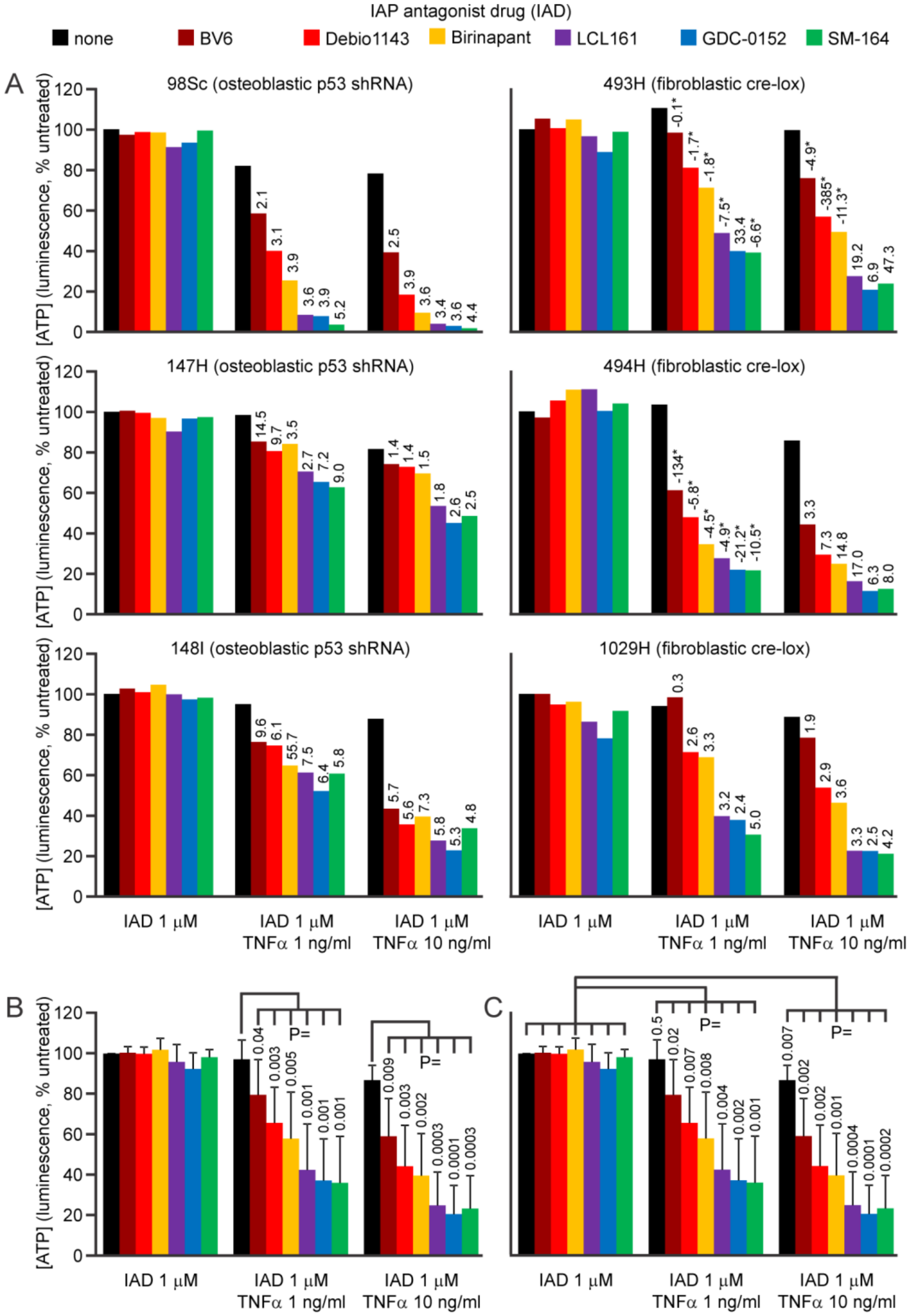

Supplementary Figure 2: IAP antagonists cooperate supra-additively with TNFα to kill murine osteosarcoma cells. Cells from the specified primary murine osteosarcomas were incubated for 48 h with the listed IAP antagonist drugs (IAD, colored columns) or media (black columns), together with murine TNFα or no death ligand. ATP levels were measured with the CellTiterGlo reagent. (A) Using the data graphed in Figure 1, the expected additive effects between the IAP antagonists and TNFα were calculated using the Bliss additivity model, whereby the additive response of both agents with individual effects T and I = T + I - (T x I). T and I were calculated as the luminescence (reflecting ATP levels) of cells treated with TNFα (T) or IAD (I) as sole agents, as a proportion of the luminescence emitted by untreated cells. The ratios of the observed to expected (additive) effects are indicated above the columns. Instances when treatment with IAP antagonists or TNFα alone yielded more luminesence than no treatment yielded some negative results. In these cases, noted with the asterisks, the expected impact of the combination was to slightly boost ATP levels in co-treated cells, but co-treatment was experimentally observed to reduce ATP levels, consistent with combined exposure killing the cells. (B, C) The average responses of cells from the six primary tumors were calculated. Error bars indicate standard deviations. Two-sided paired T-tests were used to calculate the probability that the observed differences between reponses to TNFα alone and TNFα plus each IAP antagonist (for cells from each tumour) were due to chance (B), or that the differences between responses to each IAP antagonist +/- TNFα were due to chance (C).

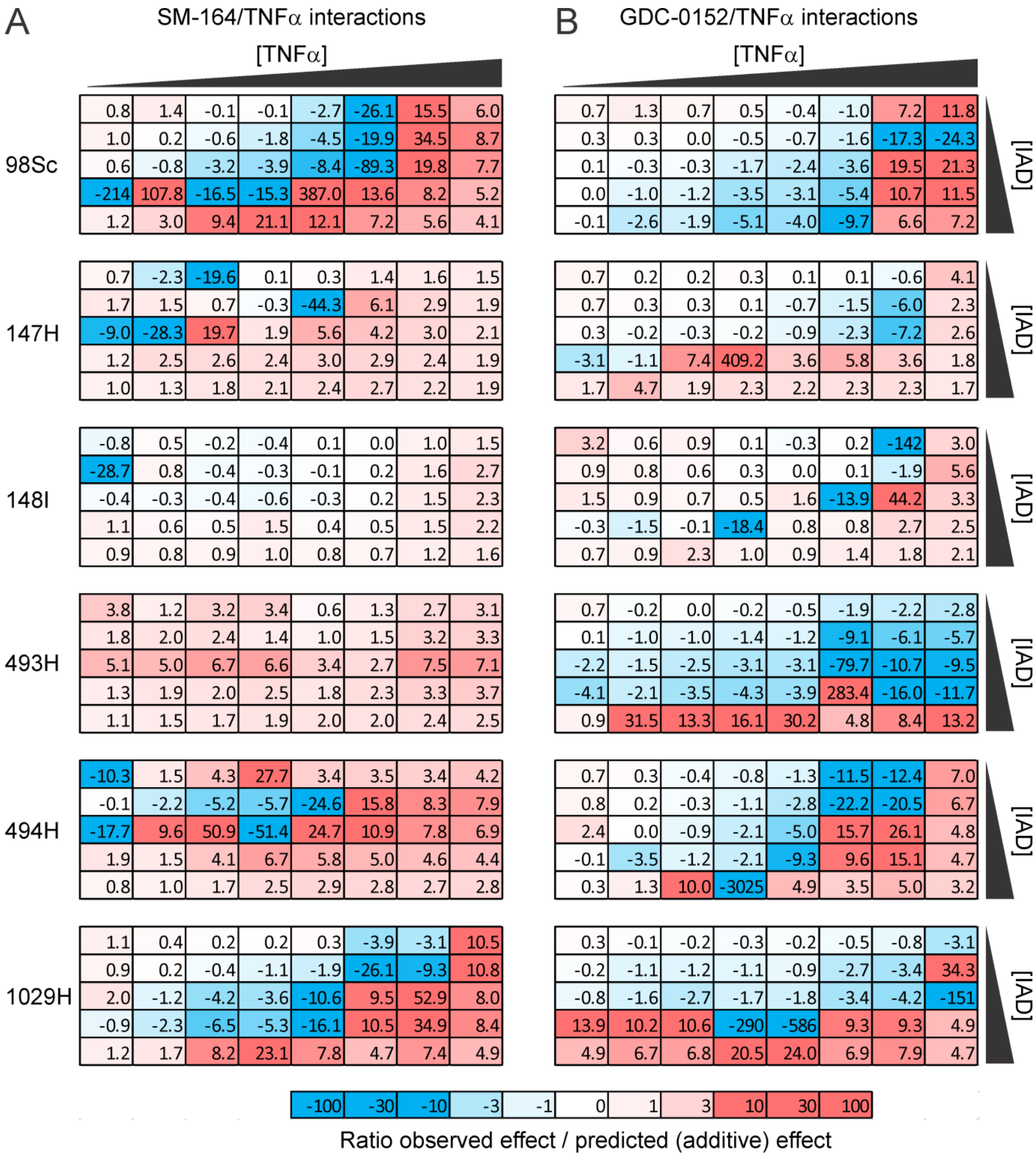

Supplementary Figure 3: SM-164 and GDC-0152 cooperate supra-additively with TNFα to kill murine osteosarcoma cells. Cells from the specified primary murine osteosarcomas were incubated for 48 h with media, 0-10 µM SM-164 (A) or GDC-0152 (B), together with 0-1 ng/ml murine TNFα or no death ligand. ATP levels were measured with the CellTiterGlo reagent. From the data graphed in Figures 2 and 3, the expected additive effects between the IAP antagonists and TNFα were calculated using the Bliss additivity model, whereby the additive response of both agents with individual effects T and I = T + I - (T x I). T and I were calculated as the luminescence (reflecting ATP levels) of cells treated with TNFα (T) or IAD (I) as sole agents as a proportion of the luminescence of untreated cells. The ratios of the observed to expected (additive) effects for each drug combination are specified in the boxes, which are color-coded as shown in the key. Instances when treatment with IAP antagonists or TNFα alone yielded more luminesence (ATP) than no treatment yielded some negative results. In these cases (blue boxes), the expected impact of the combination was to slightly boost ATP levels in co-treated cells, but co-treatment was experimentally observed to reduce ATP levels, consistent with combined exposure killing the cells.

A

98Sc

[doxorubicin] (nM)	30	100	300	1000	3000
2 way: S+T, D	1.1	1.1	1.0	1.0	1.0
2 way: S+D, T	-7.3	-15.6	3.4	1.4	1.0
2 way: T+D, S	-0.1	0.0	0.3	0.6	1.0
3 way: S, T, D	-0.09	-0.01	0.37	0.65	0.98

493H

[doxorubicin] (nM)	30	100	300	1000	3000
2 way: S+T, D	1.1	1.2	1.1	1.0	1.0
2 way: S+D, T	-34.9	7.0	2.5	1.3	1.0
2 way: T+D, S	0.0	0.2	0.4	0.8	1.0
3 way: S, T, D	-14.4	7.8	2.1	1.2	1.0

147H

[doxorubicin] (nM)	30	100	300	1000	3000
2 way: S+T, D	0.6	1.4	0.7	0.7	1.0
2 way: S+D, T	-0.2	-0.9	-5.6	1.1	1.0
2 way: T+D, S	-4.0	-1.0	-0.2	0.9	1.0
3 way: S, T, D	-0.2	-0.9	672.0	0.9	1.1

494H

[doxorubicin] (nM)	30	100	300	1000	3000
2 way: S+T, D	1.2	1.3	1.1	1.0	1.0
2 way: S+D, T	-4.6	3.4	1.8	1.2	1.0
2 way: T+D, S	0.0	0.4	0.7	0.9	1.0
3 way: S, T, D	-5.1	6.3	1.9	1.1	1.0

148I

[doxorubicin] (nM)	30	100	300	1000	3000
2 way: S+T, D	1.0	1.0	0.9	1.0	1.0
2 way: S+D, T	-2.2	-3.9	2.0	1.1	1.0
2 way: T+D, S	-0.4	-0.2	0.6	1.0	1.0
3 way: S, T, D	-3.1	-9.7	1.5	1.1	1.0

1029H

[doxorubicin] (nM)	30	100	300	1000	3000
2 way: S+T, D	1.0	1.1	1.0	1.0	1.0
2 way: S+D, T	3.5	2.2	2.0	1.3	1.1
2 way: T+D, S	0.3	0.5	0.5	0.8	1.0
3 way: S, T, D	3.4	2.7	1.8	1.2	1.1

B

98Sc

[doxorubicin] (nM)	300	1000	3000
2 way: S+T, D	1.4	1.7	1.1
2 way: S+D, T	7.9	4.2	1.0
2 way: T+D, S	0.2	0.2	0.9
3 way: S, T, D	5.4	0.7	1.0

key

-100	-1	0	1	3	10	30

Supplementary Figure 4: SM-164 plus TNFα cooperate additively with doxorubicin to kill murine osteosarcoma cells. Osteosarcoma cells from the specified murine tumors were incubated as described in the legend to Figure 5. ATP levels in the cells were then measured using the CellTiterGlo kit (A) or the proportions of 98Sc cells staining with Annexin-V-FITC were assayed using flow cytometry (B). From the data graphed in Figure 5, the expected additive effects between the drugs were calculated using the Bliss additivity model. Expected interactions between a pair of drugs (P) with a third drug (3rd) were calculated using this equation: 2 way expected interaction = P + 3rd - (P x 3rd). Expected interactions between all three drugs based on their single effects were calculated using this equation: 3 way expected interaction = S + T + D - (S x T) – (S x D) – (T x D). The ratios of the observed to expected (additive) effects for each drug combination are specified in the boxes, which are color-coded as shown in the key. Instances when treatment with SM-164 or TNFα alone yielded more luminesence (ATP) than no treatment yielded some negative results. In these cases (blue boxes), the expected impact of the combination was to slightly boost ATP levels in co-treated cells, but co-treatment was experimentally observed to reduce ATP levels, consistent with combined exposure killing the cells.

A

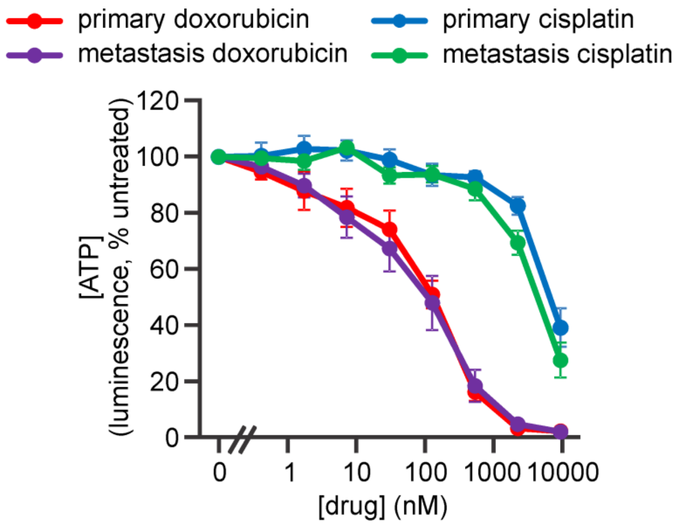

B

	IC$_{50}$ (nM, +/- SEM)			
	doxorubicin		cisplatin	
mouse	primary	metastasis	primary	metastasis
98	41	42	196424	3681
1029	278	125	9610	5435
147	295	683	7507	10678
148	274	105	4374	5459
493	366	234	7647	5174
494	331	540	6200	3177
paired, two-tailed P value	0.808		0.349	

Supplementary Figure 5: Primary and metastatic osteosarcoma cells respond similarly to chemotherapy drugs. Cells from primary or metastatic tumors were incubated in the media containing no drugs or the indicated concentrations of doxorubicin or cisplatin for 48 h, then ATP levels were measured. (A) Using the data graphed in Figure 9, the mean responses of cells from six primary and six metastatic tumors to doxorubicin and cisplatin were calculated (+/- SEM). (B) The doses of drugs that reduced the ATP levels by half were calculated. Two-sided paired T-tests were performed to estimate the probability that the differences in drug sensitivities of primary versus metastatic cells occurred due to chance.

Supplementary table 1: P values analysing clonogenicity data from Figure 4. Two sided paired T tests were calculated to analyse the probability that the observed differences in clonogenic impact of combined SM-164+TNFα exposure versus single drug and no drug treatments were due to chance.

**24 h exposure**	**98Sc**	**494H**	**1029H**
SM-164+TNFα versus untreated	0.019	0.022	0.002
SM-164+TNFα versus SM-164 alone	0.042	0.045	0.005
SM-164+TNFα versus TNFα alone	0.038	0.028	0.003
**Continuous exposure**			
SM-164+TNFα versus untreated	0.005	0.023	0.002
SM-164+TNFα versus SM-164 alone	0.002	0.042	0.004
SM-164+TNFα versus TNFα alone	0.009	0.042	0.008
**SM-164+TNFα**			
24h versus continuous exposure	0.179	0.061	0.061

Supplementary table 2: Raw clonogenicity data from Figure 4

		98Sc colonies per 300 cells seeded			494H colonies per 100 cells seeded			1029H colonies per 100 cells seeded		
		rep 1	rep 2	rep 3	rep 1	rep 2	rep 3	rep 1	rep 2	rep 3
**24 h exposure**	untreated	25	32	30	60	47	47	39	46	53
	TNFα	24	30	30	59	50	46	40	47	52
	SM-164	25	30	31	60	45	45	38	47	49
	SM-164 + TNFα	13	22	14	43	34	37	20	30	35
**Replaced every 48h**	untreated	30	36	34	55	54	48	45	48	50
	TNFα	29	36	32	50	52	42	40	48	51
	SM-164	28	32	32	55	45	44	48	44	49
	SM-164 + TNFα	8	9	12	19	20	27	11	13	11

Supplementary table 3: P values analysing drug sensitivity data from Figure 8. Two sided paired T tests were calculated to analyse the probability that the observed differences in ATP levels following exposure to SM-164+TNFα in the presence and absence of Q-VD-OPh or necrostatin could be due to chance.

Tumour	SM-164 + TNFα versus SM-164 + TNFα + *Q-VD-OPh*	SM-164 + TNFα versus SM-164 + TNFα + *necrostatin*
98Sc	0.456	0.005
1029H	0.020	0.058
493H	0.038	0.058
494H	0.739	0.040
147H	0.486	0.306
148I	0.498	0.080
98L	0.112	0.133
1029LV	0.013	0.002
493L	0.296	0.119
494L	0.038	0.010
147L	0.024	0.001
148L	0.181	0.262
